# Fc*γ*RIV is required for IgG2c mediated enhancement of RBC alloimmunization

**DOI:** 10.3389/fimmu.2022.972723

**Published:** 2022-09-13

**Authors:** Annie Qiu, Anabel Miller, Flavia Dei Zotti, Manjula Santhanakrishnan, Jeanne E. Hendrickson, Maria Tredicine, Sean R. Stowell, Chance John Luckey, James C. Zimring, Krystalyn E. Hudson

**Affiliations:** ^1^ Department of Pathology and Cell Biology, Columbia University Irving Medical Center, New York, NY, United States; ^2^ Department of Laboratory Medicine, Yale University School of Medicine, New Haven, CT, United States; ^3^ Department of Translational Medicine and Surgery, Section of General Pathology, Università Cattolica del Sacro Cuore, Rome, Italy; ^4^ Department of Pathology, Brigham and Women’s Hospital, Harvard Medical School, Boston, MA, United States; ^5^ Department of Pathology, University of Virginia, Charlottesville, VA, United States; ^6^ Carter Immunology Center, University of Virginia, Charlottesville, VA, United States; ^7^ Department of Pathology, University of Virginia School of Medicine, Charlottesville, VA, United States

**Keywords:** Fc receptor, antibody, red blood cell, alloantibody, antibody mediated enhancement

## Abstract

Passive immunization with anti-D can prevent maternal alloimmunization to RhD thereby preventing hemolytic disease of the fetus and newborn. Unexpectedly, anti-D fails in some cases and some monoclonal anti-D preparations paradoxically enhances alloimmunization. The underlying mechanisms modulating humoral alloimmunization by anti-D are unknown. We previously reported that IgG antibody subclasses differentially regulate alloimmunity in response to red blood cell (RBC) transfusions in a mouse model; in particular, IgG2c significantly enhanced RBC alloantibody responses. Initial mechanistic studies revealed that IgG2c:RBC immune complexes were preferentially consumed by the splenic dendritic cell (DC) subsets that play a role in RBC alloimmunization. The deletion of activating Fc-gamma receptors (FcγRs) (i.e., FcγRI, FcγRIII, and FcγRIV) on DCs abrogated IgG2c-mediated enhanced alloimmunization. Because DCs express high levels of FcγRIV, which has high affinity for the IgG2c subclass, we hypothesized that FcγRIV was required for enhanced alloimmunization. To test this hypothesis, knockout mice and blocking antibodies were used to manipulate FcγR expression. The data presented herein demonstrate that FcγRIV, but not FcγRI or FcγRIII, is required for IgG2c-mediated enhancement of RBC alloantibody production. Additionally, FcγRI is alone sufficient for IgG2c-mediated RBC clearance but not for increased alloimmunization, demonstrating that RBC clearance can occur without inducing alloimmunization. Together, these data, combined with prior observations, support the hypothesis that passive immunization with an RBC-specific IgG2c antibody increases RBC alloantibody production through Fc*γ*RIV ligation on splenic conventional DCs (cDCs). This raises the question of whether standardizing antibody subclasses in immunoprophylaxis preparations is desirable and suggests which subclasses may be optimal for generating monoclonal anti-D therapeutics.

## Introduction

Prevention of maternal alloimmunization to RhD by passive immunization with polyclonal anti-D (i.e., immunoprophylaxis) dramatically decreased rates of hemolytic disease of the fetus and newborn. Nonetheless, some anti-D preparations, under certain conditions, paradoxically enhance alloimmunization (1, 2). The underlying mechanisms by which anti-D modulates humoral alloimmunization remain unknown, but different hypotheses involve antigen modulation, increased RhD^+^ red blood cell (RBC) clearance, preferential ligation of inhibitory Fc receptors on B cells, and/or steric hindrance/antigen masking (3–5). Although polyclonal anti-D preparations offer numerous advantages (e.g., polyvalent specificity), several limitations apply, including the number of human volunteers required, highly variable time-to-response conversion to produce anti-D alloantibodies, and batch-to-batch variations (5, 6). Thus, there is a considerable need to develop a monoclonal antibody approach, which would provide a cost-effective, scalable option that offers batch-to-batch consistency. Although many monoclonal anti-D antibodies have been tested, some enhanced RBC alloantibody responses whereas others suppressed (7–9). Additionally, there was discordance between RhD^+^ RBC clearance and immunoprophylaxis among the anti-D monoclonal antibodies evaluated (8), challenging the explanation that anti-D works by clearing RhD^+^ RBCs. Recently, two new monoclonal anti-D antibodies demonstrated efficacy in preventing RhD alloimmunization in pregnant women (10, 11), although additional studies with a larger number of participants and re-exposure to RhD+ RBCs are required to fully evaluate the efficacy of these new therapeutics.

Human polyclonal anti-D consists of four IgG subclasses: IgG1, IgG2, IgG3, and IgG4, each with distinct effector functions, including complement fixation and ligation of specific Fc-gamma (*γ*) receptors (Fc*γ*Rs) (12). To gain insight into how different subclasses modulate immune responses, we previously reported the generation of monoclonal antibodies specific for model RBC alloantigens in mice, and the generation of panels of IgG switch variants for each murine IgG subclass: IgG1, IgG2a/c, IgG2b, and IgG3 (13, 14). Across these models, passive immunization with the RBC-specific IgG2c subclass consistently enhanced RBC alloimmunization rates and alloantibody levels (13–15). In initial mechanistic studies, IgG2c:RBC immune complexes were preferentially consumed by splenic CD11c^+^CD11b^+^ and CD11c^+^CD8^+^ conventional dendritic cells (cDCs), which are known to be important in immune responses to RBCs (13, 16, 17). In addition, the IgG2c-mediated enhancement of alloimmunization depended on specific Fc*γ*R expression; that is, deleting the common *γ* chain required for expression of all activating Fc*γ*Rs (i.e., Fc*γ*RI, Fc*γ*RIII, Fc*γ*RIV) on splenic cDCs abrogated alloantibody production (13). By cellular surface plasmon resonance (cSPR), Fc*γ*RI and Fc*γ*RIV have been shown to have strong affinity for IgG2c (14). Based on prior observations demonstrating the involvement of Fc*γ*RIV in immune responses mediated by IgG2a (i.e., a genetic variant of IgG2c) (18), in initiating autoimmune responses (19, 20), and in CD4^+^ T cell priming by cDCs (21), we hypothesized that Fc*γ*RIV is required for IgG2c-mediated enhanced alloimmunization to RBC antigens.

Herein, we report that Fc*γ*RIV is required for enhanced RBC alloimmunization following passive immunization with IgG2c; that is, inhibiting Fc*γ*RIV abrogated RBC alloantibody production. Fc*γ*RIII was not required and Fc*γ*RI was not sufficient for enhancing alloimmune responses. Unexpectedly, Fc*γ*RI was not sufficient for increased alloimmunization but was alone sufficient for IgG2c-mediated RBC clearance from the circulation, providing further evidence that RBC clearance need not be directly linked to alloantibody production. Together, these data provide additional information into which Fc*γ*Rs are responsible for that IgG2c-mediated enhancement of RBC alloimmunization.

## Materials and methods

### Mice

B6 (C57BL/6NCrl, stock #027) were purchased from Charles River and Fc*γ*RIII^-/+^ (B6.129P2-Fcgr3^tm1Jsv^/J; stock #003171) were purchased from The Jackson Laboratory; these mice were generated on a 129/B6 mixed background and backcrossed to C57BL/6 for 7 generations. Thus, to control for genetic background differences, Fc*γ*RIII^-/+^ mice were bred to generate Fc*γ*RIII^+/+^ mice for control recipients. For experiments, Fc*γ*RIII^-/+^ mice were bred to generate Fc*γ*RIII^-/-^ mice. Mice expressing an RBC-specific triple fusion protein consisting of hen egg lysozyme, ovalbumin, and the human blood group molecule Duffy (HOD) were generated, as previously described (22). All mice were maintained in a pathogen-free environment on standard rodent chow and water in a light and temperature-controlled environment. Unless otherwise stated, mice were 8-24 weeks old and both male and female animals were used. All protocols used were approved by the Columbia University Irving Medical Campus Institutional Animal Care and Use Committee (IACUC).

### Treatment of mice

Anti-HOD mAb IgG2c antibody (1 *μ*g/mouse, which recognizes an epitope common to Duffy (Fy^a^ and Fy^b^) blood group molecule contained within the HOD antigen) was passively infused into recipients 2 hours before an RBC transfusion, as previously described (13). Transfusions consisted of 50*μ*L of leukoreduced CellTrace-CFSE (ThermoFisher) labeled HOD packed RBCs (pRBCs) and 50*μ*L of leukoreduced CellTrace-Far Red (ThermoFisher) labeled B6 pRBCs diluted to a 20% hematocrit with PBS. In some experiments, recipients were infused with 200*μ*g of the purified monoclonal anti-mouse CD16.2 (Fc*γ*RIV) blocking antibody (Biolegend, Clone 9e9) or Armenian hamster IgG isotype control with anti-glutathione S-transferase specificity (BioXCell #BE0260, Clone PIP) 30 minutes prior to passive immunization. To evaluate the specificity of the Fc*γ*RIV blocking antibody, spleens were collected and stained with antibodies to delineate leukocyte subsets and Fc receptor expression, as previously described (13). Post-transfusion survival of allogeneic RBCs, and subsequent alloantibody production, were performed, as previously described (13).

### Detection of alloantibodies by flow crossmatch

Sera was collected from experimental mice, diluted 1:100 and added to HOD target RBCs or B6 control target RBCs in FACS buffer (phosphate-buffered saline + 0.2mg/mL bovine serum albumin + 0.9mg/mL ethylenediaminetetraacetic acid). RBCs and sera were incubated for 20 minutes at room temperature, washed three times with FACS buffer and then stained with 1:100 goat anti-mouse immunoglobulins (IgM + IgG + IgA) conjugated to APC as secondary detection reagent. To determine the isotype and subclass of alloantibodies, we used directly-conjugated antibodies against IgM, IgG1, IgG2b, IgG2c, and IgG3. Samples were incubated at 4°C for 30 minutes and then washed three times with FACS buffer prior to analysis with a flow cytometer. To calculate the adjusted MFI, the sera incubated with B6 RBCs (negative control/background signal) was subtracted from the signal from sera incubated with HOD RBCs. The only difference between HOD and B6 RBCs is the expression of the HOD antigen, which is confirmed with PUMA6 and goat anti-mouse immunoglobulin-APC staining.

### RBC labeling

HOD and B6 whole blood was collected into 14% CPDA-1 from donor animals by retro-orbital exsanguination and leukoreduced with an Acrodisc PSF syringe filter (Pall Life Sciences). For CellTrace-CFSE labeling, 10mL of CellTrace-CFSE (5 *μ* M) was added for every 1mL of pRBCs. For CellTrace-Far Red, 2mL of CellTrace-Far Red (5 *μ* M) was added for every 1mL of pRBCs. pRBCs were added directly into either working solutions of CellTrace-CFSE or CellTrace-Far Red, swirled to mix, and incubated at 37°C in the dark for 20 minutes, inverting after 10 minutes. RBCs were washed twice with PBS and resuspended at 20% hematocrit in PBS. Labelled HOD and B6 RBCs were then mixed at a 1:1 ratio. To determine pre-transfusion ratio, an aliquot of mixed RBCs was analyzed by flow cytometry.

### Leukocyte isolation and FcγR staining

Spleens from mice were collected into complete RPMI, collagenase digested, and filtered, as previously described (13, 23). Single cells were washed with FACS buffer, RBCs were lysed, and the remaining cells were stained with antibodies to delineate leukocyte subsets (**Supplemental Table 1**). To enable detection of FcγRs, cells were stained with antibodies against FcγRI, FcγRII, FcγRIII, and FcγRIV; to prevent potential steric hindrance, cells were stained for an individual FcγR to evaluate expression levels. Antibodies specific for Thy1.2, Ter119, PDCA1, CD115, Ly6G, CD11c, F4/80, and CD8 were purchased from ThermoFisher those recognizing CD19, CD11b, FcγRI, FcγRII, and FcγRIV were purchased from BioLegend. An antibody to FcγRIII was purchased from Bio-Rad. All staining was performed in FACS buffer, and cells were interrogated on an Attune NxT flow cytometer (ThermoFisher), and data analyzed with FlowJo software.

### Statistical analysis

Statistical analysis was performed with a repeated measures 2-way ANOVA or a one-way ANOVA with Tukey’s multiple comparisons test; p<0.05 was considered significant. Analyses were performed using Prism, version X (GraphPad Software, Inc.).

## Results

### Inhibiting Fc*γ*RIV abrogates enhanced RBC alloantibody production induced by passive immunization with IgG2c

**Figure 1:**
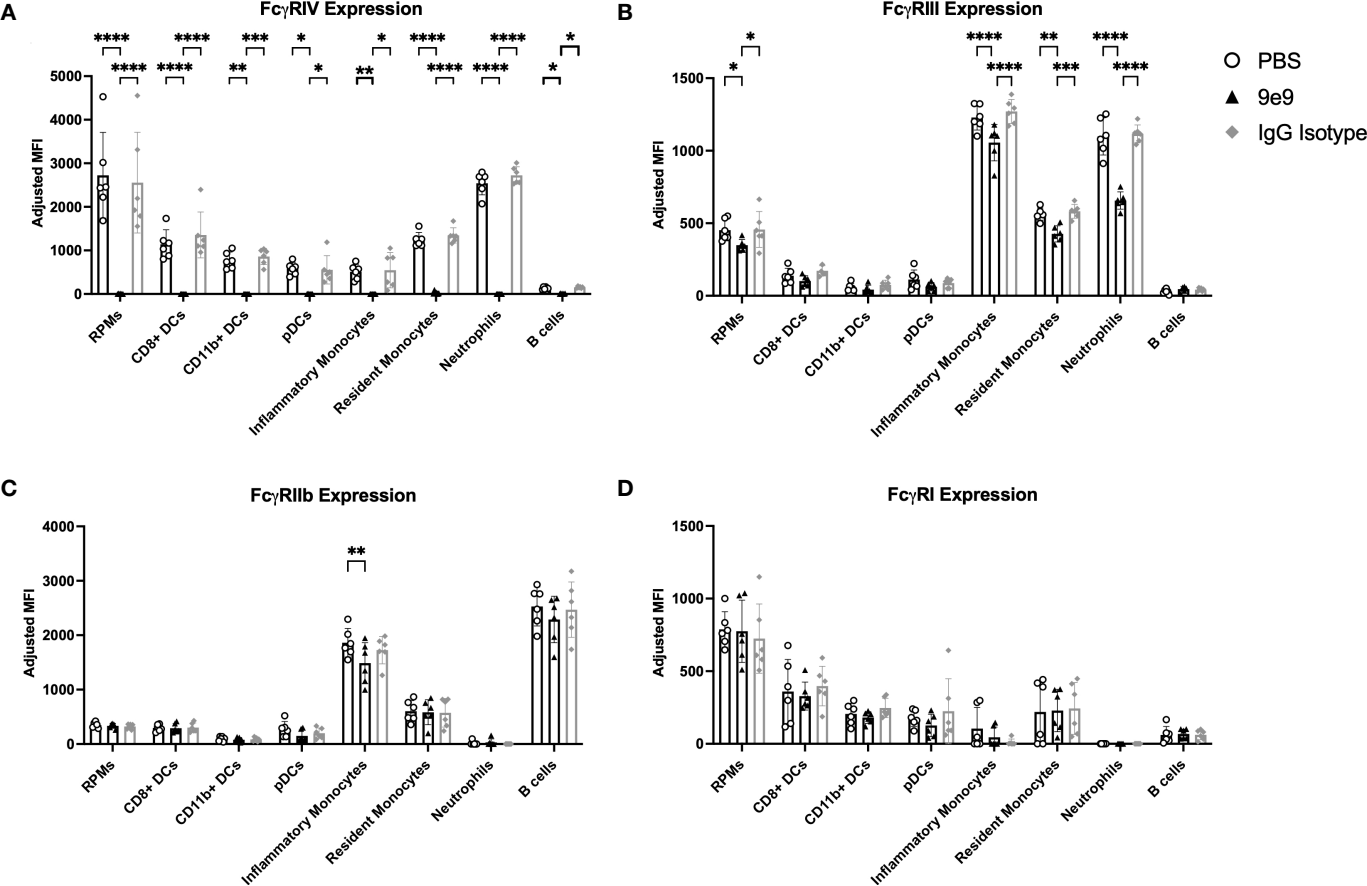
Anti-FcγRIV (clone 9e9) inhibits FcγRIV. B6 recipient mice received an infusion of 200μg of anti-FcγRIV clone 9e9, IgG isotype control, or PBS. Spleens were collected 30 minutes later and processed into single cell suspensions. Splenocytes were stained with antibodies to delineate cell subsets and expression of **(A)** FcγRIV, **(B)** FcγRIII, **(C)** FcγRIIb, or **(D)** FcγRI. The mean fluorescence intensity (MFI) for each individual FcγR was calculated; a fluorescence minus one (FMO) was used as a negative control. To determine the adjusted MFI, the MFI of the FMO was subtracted from the MFI of the stain containing the antibody for the FcγR. The following phenotypes were used to delineate cell subsets: red pulp macrophages (RPMs): CD11c^−/lo^CD11b^−/lo^F4/80^+^; CD8^+^ DCs: CD11c^hi^CD11b^−^CD8^+^; CD11b^+^ DCs: CD11c^hi^CD11b^+^CD8^−^; plasmacytoid DCs (pDCs): PDCA1^+^CD11c^int^Ly6C^hi^; inflammatory monocytes: CD11c^−/lo^CD11b^+^Ly6G^−^Ly6C^hi^CD115^+^; resident monocytes: CD11c^−/lo^CD11b^+^Ly6G^−^Ly6C^lo^CD115^−^; neutrophils: CD11c^−/lo^CD11b^+^Ly6G^+^ and a high side scatter. Data shown are an aggregate of 2 independent experiments with 3 mice per group. Statistical analysis was performed with a one-way ANOVA with Tukey’s multiple comparisons test; ****p<0.0001, ***p<0.001, **p<0.05, *p<0.05.

Passive immunization with anti-HOD mAb IgG2c was previously shown to enhance alloantibody production following transfusion with RBCs expressing the HOD alloantigen (13). This enhancement required expression of activating Fc*γ*Rs on CD11c^+^CD8^+^ and CD11c^+^CD11b^+^ cDC subsets (13), although which individual Fc*γ*R(s) were required remained unknown. To elucidate specific Fc*γ*Rs, knockout mice and blocking antibodies were used. Fc*γ*RIV activity was inhibited by infusion of an anti-CD16.2 antibody (clone 9e9; herein referred to as “9e9”). Nonspecific effects were controlled for using either an IgG isotype control or PBS. 9e9 treatment inhibited detection of Fc*γ*RIV on all splenic antigen presenting cell subsets evaluated, effectively reducing the staining signal to background levels (**Figure 1A**). Unexpectedly, 9e9 infusion also led to decreased expression of Fc*γ*RIII on red pulp macrophages, inflammatory monocytes, resident monocytes, and neutrophils (**Figure 1B**) and decreased expression of inhibitory Fc*γ*RIIb on inflammatory monocytes (**Figure 1C**). No significant differences were observed with Fc*γ*RI expression (**Figure 1D**). Absolute counts of each cell subset revealed 9e9 infusion blocked Fc*γ*RIV but did not promote cell death (**Supplemental Figure 1**). In contrast, levels of each individual Fc*γ*R were similar between the IgG isotype control-treated and PBS-treated animals.

**Figure 2:**
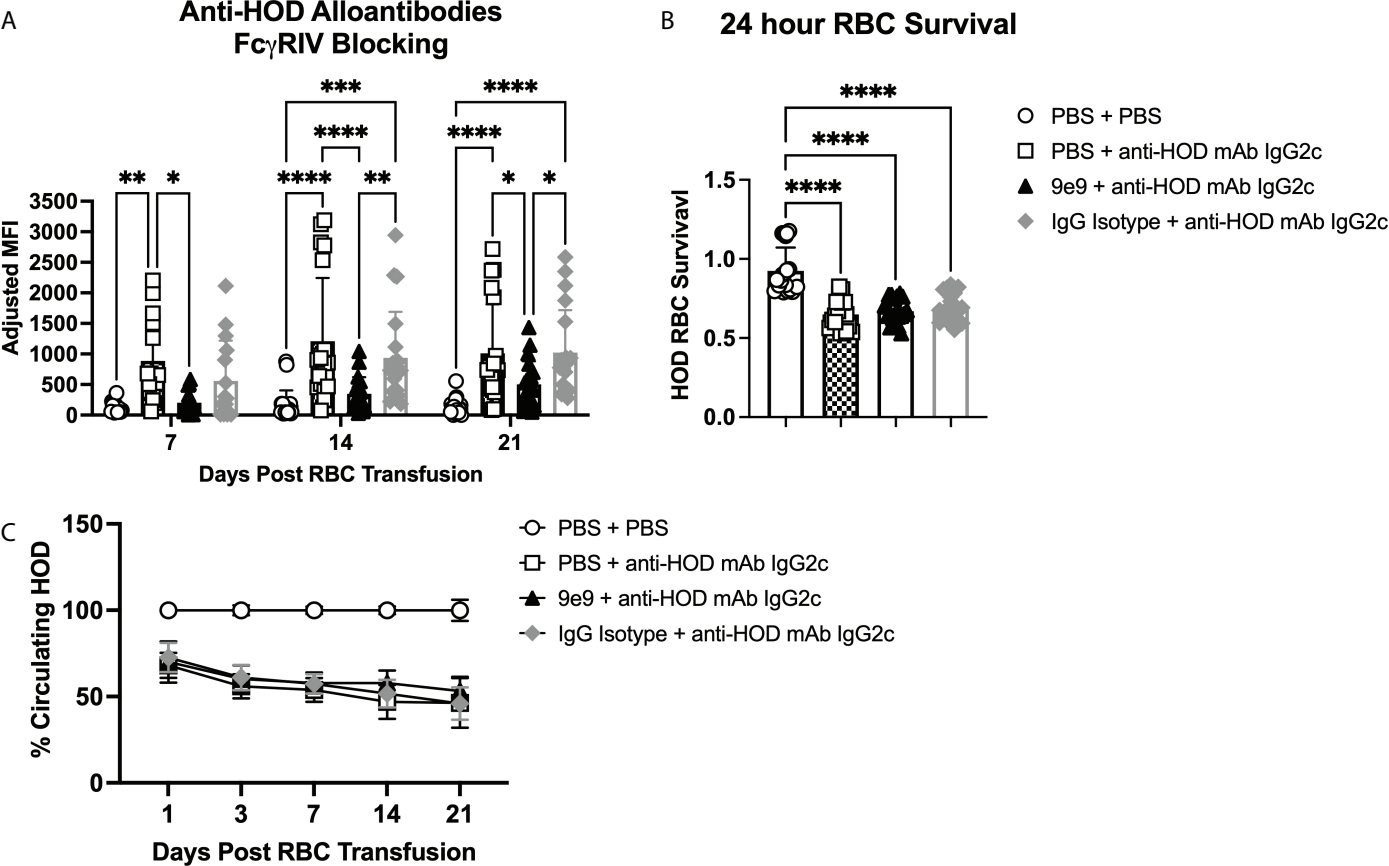
RBC alloantibody production requires FcγRIV. B6 recipient mice were passively immunized with 1μg of anti-HOD IgG2c followed by an RBC transfusion 2 hours later. Some animals received an infusion of 1μg of 9e9, an anti-FcγRIV blocking antibody, or IgG isotype control 30 minutes prior to passive immunization. Sera was collected weekly and **(A)** analyzed for anti-HOD alloantibodies by flow crossmatch. To assess RBC clearance, survival of HOD RBCs was determined at **(B)** 24 hours and **(C)** over a 3-week time course. Data shown are cumulative of 4 independent experiments with 4-5 mice per group. Statistical analysis was performed with a repeated measures 2-way ANOVA or a one-way ANOVA with Tukey’s multiple comparisons test, ****p<0.0001, ***p<0.001, **p<0.05, *p<0.05, comparisons without significance are not shown.

To assess whether Fc*γ*RIV inhibition affected RBC alloimmunization, recipient B6 mice were infused with 9e9, an IgG isotype control antibody, or PBS 30 minutes before passive immunization with anti-HOD mAb IgG2c or PBS control. Each recipient was then transfused 2 hours later with a 1:1 mix of fluorescently-labeled allogeneic HOD and syngeneic B6 RBCs (**Supplemental Figure 2**). Consistent with prior observations (13), B6 mice passively immunized with anti-HOD mAb IgG2c had significantly higher anti-HOD alloantibody production throughout the 21-day time course, as compared to (PBS + PBS) control mice (**Figure 2A**). In contrast, 9e9 pre-treatment significantly reduced anti-HOD alloantibodies over the 21-day time course, as compared to (PBS + anti-HOD mAb IgG2c) treated animals. No significant differences were observed between the (PBS + PBS) and the (9e9 + anti-HOD mAb IgG2c) treated groups, demonstrating that inhibiting Fc*γ*RIV abrogated enhanced RBC alloantibody production. Additionally, levels of anti-HOD alloantibody production were similar between anti-HOD mAb IgG2c groups pre-treated with PBS or the IgG isotype control, providing evidence that reduction in alloantibody production was specific to 9e9 recognizing its epitope, and not due to nonspecific antibody effects. Analysis of IgM and IgG subclasses showed similar a similar trend as total immunoglobulins (**Supplemental Figure 3**).

We previously reported that passive immunization with IgG2c induces significant allogeneic RBC clearance, which is mitigated by decreased expression of activating Fc*γ*RI, Fc*γ*RIII, and Fc*γ*RIV (13). To test whether inhibiting Fc*γ*RIV affected HOD RBC clearance, post-transfusion survival was measured over a the 3-week time course. Consistent with prior observations (13), HOD RBC survival at 24-hours was significantly reduced in (PBS + anti-HOD mAb IgG2c) treated animals compared to (PBS + PBS) controls with no anti-HOD mAb IgG2c (**Figure 2B**). However, no differences in clearance were observed between groups that received 9e9 or an IgG isotype control prior to anti-HOD IgG2c mAb. To evaluate potential long-term differences between experimental groups, HOD RBC survival was normalized to the (PBS + PBS) treated control group. No significant differences in HOD RBC survival were noted between groups passively immunized with anti-HOD IgG2c mAb (**Figure 2C**). Together, these data demonstrate that, although Fc*γ*RIV is required for IgG2c-mediated alloantibody production, but it is not required for allogeneic RBC clearance.

### Fc*γ*RIII is not required and Fc*γ*RI is not sufficient for enhanced RBC alloantibody production

**Figure 3:**
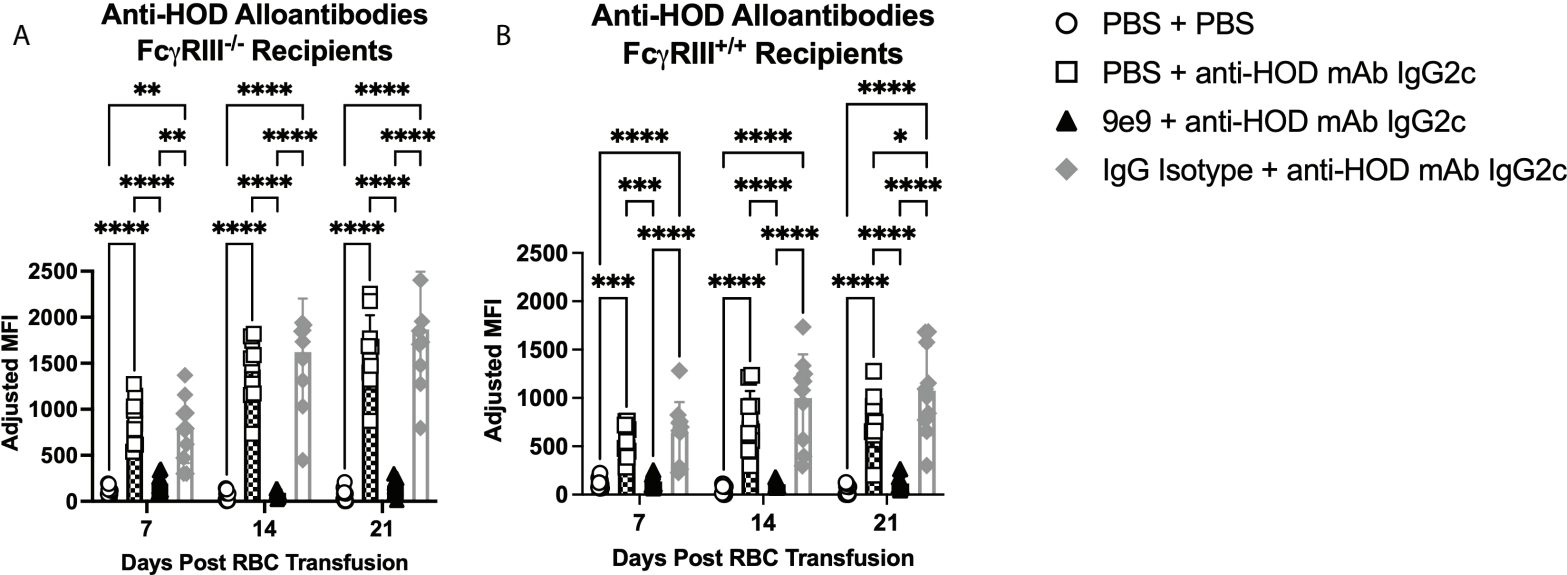
Neither FcγRI nor FcγRIII are sufficient for enhanced alloantibodies with IgG2c. Recipient mice were passively immunized with 1μg of anti-HOD IgG2c followed by an RBC transfusion 2 hours later. Some animals received an infusion of 1μg of 9e9 or IgG isotype control 30 minutes prior to passive immunization. Sera was collected from **(A)** FcγRIII^-/-^ and **(B)** FcγRIII^+/+^ recipient mice weekly and analyzed for anti-HOD alloantibodies by flow crossmatch. Data shown are cumulative of 2 independent experiments with 5 mice per group. Statistical analysis was performed with a repeated measures 2-way ANOVA with Tukey’s multiple comparisons test; ****p<0.0001, ***p<0.001, **p<0.05, *p<0.05, comparisons without significance are not shown.

To test the roles of Fc*γ*RIII and Fc*γ*RI in IgG2c-mediated enhanced alloantibody production, knockout mice and 9e9 were used together. Passive immunization with anti-HOD IgG2c mAb followed by RBC transfusion into Fc*γ*RIII^-/-^ animals induced significantly increased anti-HOD alloantibody levels at all time points, as compared to (PBS + PBS) treated control animals (**Figure 3A**). Because Fc*γ*RIII^-/-^ mice were generated on a 129/B6 mixed background, Fc*γ*RIII^+/+^ replete mice were used as controls to isolate the contribution of Fc*γ*RIII in alloimmune responses. These data demonstrate that FcγRIII is not required for IgG2c-mediated enhanced RBC alloimmunization. To test whether Fc*γ*RI alone was sufficient for enhancing alloantibodies with IgG2c, FcγRIII^-/-^ mice were pre-treated with 9e9 or IgG isotype control. Pre-treating FcγRIII^-/-^ mice with 9e9 (thereby eliminating both Fc*γ*RIII and Fc*γ*RIV, and leaving Fc*γ*RI signaling intact) significantly reduced anti-HOD alloantibodies to levels comparable to (PBS + PBS) treated control mice (**Figure 3A**, black triangles). RBC alloantibodies were not reduced in the IgG isotype control group (**Figure 3A**, gray diamonds). Thus, Fc*γ*RI is not sufficient for increasing alloantibody production upon passive immunization with anti-HOD IgG2c mAb. To exclude background genetics as a potential confounder, experiments were performed in Fc*γ*RIII^+/+^ mice, in parallel, and similar trends were observed as in Fc*γ*RIII^-/-^ mice (**Figure 3B**).

### Fc*γ*RI is sufficient and Fc*γ*RIII is not required for mediating RBC clearance by passive immunization with IgG2c

**Figure 4:**
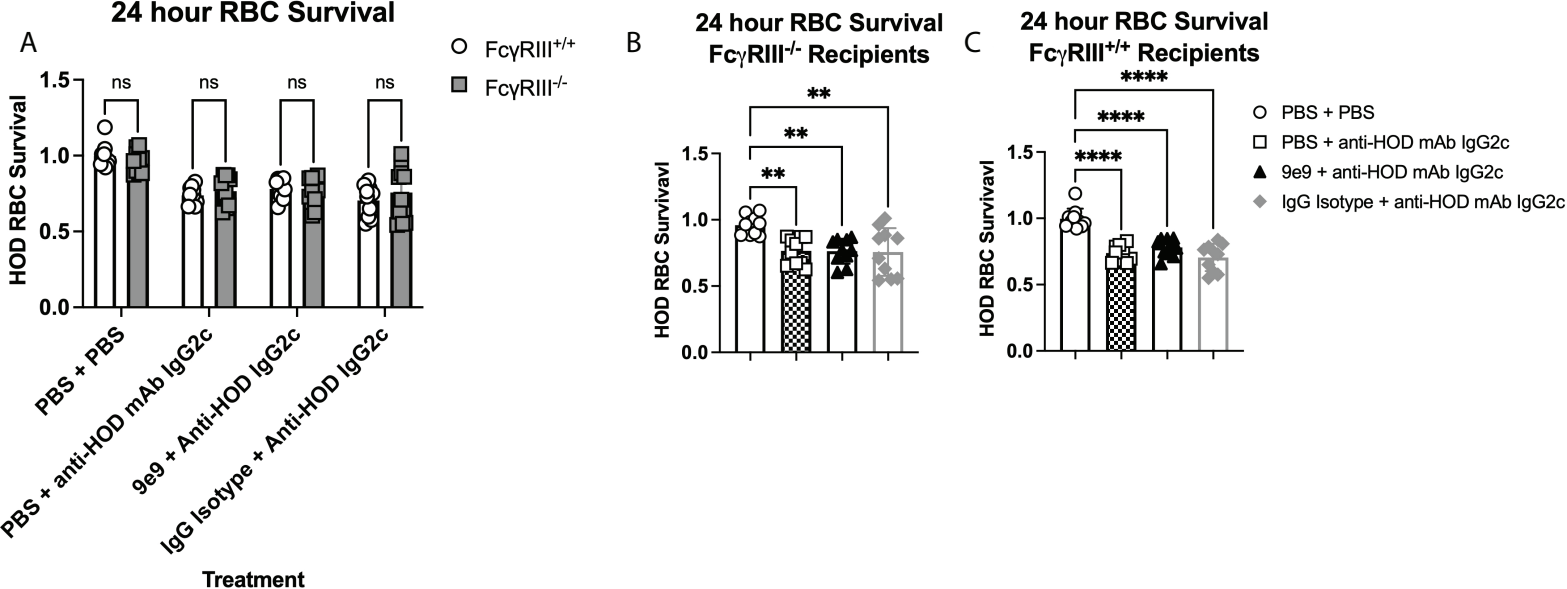
FcγRI is sufficient to mediate RBC clearance with IgG2c. Recipient mice were passively immunized with 1μg of anti-HOD IgG2c followed by an RBC transfusion 2 hours later. Some animals received an infusion of 1μg of 9e9 or IgG isotype control 30 minutes prior to passive immunization. To assess RBC clearance, 24 hour survival of HOD RBCs was determined in **(A)** all treatment groups and recipients, or between treatment groups within **(B)** FcyRIII^-/-^ , or **(C)** FcyRIII^+/+^ recipient mice. Data shown are cumulative of 2 independent experiments with 5 mice per group. Statistical analysis was performed with a one-way ANOVA with Tukey’s multiple comparisons test; ****p<0.0001, **p<0.05, ns, not significant.

Passive immunization with anti-HOD IgG2c mAb leads to rapid HOD RBC clearance (**Figures 2B, C**). Similar levels and kinetics of clearance were observed upon blocking Fc*γ*RIV with 9e9 (**Figure 2B**), suggesting clearance is mediated by Fc*γ*RI, Fc*γ*RIII, or both. To evaluate Fc*γ*RIII-mediated clearance of allogeneic HOD RBCs by anti-HOD IgG2c mAb, 24-hour survival was determined in Fc*γ*RIII^-/-^ and Fc*γ*RIII^+/+^ mice. HOD RBC clearance by IgG2c was equivalent in Fc*γ*RIII^+/+^ and Fc*γ*RIII^-/-^ mice (**Figure 4A**). Thus, Fc*γ*RIII is not required for IgG2c-mediated clearance of HOD RBCs. To evaluate Fc*γ*RI function alone, Fc*γ* RIV was inhibited in Fc*γ*RIII^-/-^ animals; this approach leaves only Fc*γ*RI signaling intact. No significant change was observed in HOD RBC clearance between 9e9 or IgG isotype control treated Fc*γ*RIII^-/-^ animals (**Figure 4B**), demonstrating that, Fc*γ*RI expression is alone sufficient for HOD RBC clearance by anti-HOD IgG2c mAb; similar results were observed in Fc*γ*RIII^+/+^ control mice (**Figure 4C**). No significant differences in HOD RBC survival were noted between groups passively immunized with anti-HOD IgG2c mAb in Fc*γ*RIII^+/+^ or Fc*γ*RIII^-/-^ mice (**Supplemental Figure 4**). Together, these data demonstrate that Fc*γ*RI is sufficient for IgG2c-mediated RBC clearance, and that Fc*γ*RIII is not required.

## Discussion

A murine model of RBC alloimmunization was used to test the hypothesis that Fc*γ*RIV is required for enhanced alloantibody production upon passive immunization with IgG2c. Inhibiting Fc*γ*RIV with 9e9, an antibody that blocks its binding site, reduced RBC alloantibody production to background levels. Passive immunization with anti-HOD IgG2c mAb into Fc*γ*RIII^-/-^ mice, which still express Fc*γ*RI and Fc*γ*RIV, enhanced RBC alloantibody levels, demonstrating that Fc*γ*RIII was not required. Likewise, inhibiting Fc*γ*RIV in Fc*γ*RIII^-/-^ animals, but leaving Fc*γ*RI signaling intact, failed to induce RBC alloantibodies, indicating that Fc*γ*RI was not sufficient for enhanced alloantibody production. In parallel, Fc*γ*RI was shown to be sufficient for anti-HOD IgG2c mAb-mediated RBC clearance, demonstrating that the mechanisms of IgG2c-mediated clearance and alloantibody production involve distinct Fc*γ*Rs. These data, together with our prior publication (13), provide evidence that enhanced alloantibody production following passive immunization with antigen-specific IgG2c and transfusion of the corresponding antigen-expressing RBCs, requires Fc*γ* RIV expression on splenic cDCs.

The reliance on Fc*γ*RIV for IgG2a-mediated effector functions has been demonstrated in several models including B cell depletion, phagocytosis of platelets, and autoimmune responses such as autoimmune hemolytic anemia, rheumatoid arthritis, and nephritis (18, 24–27). Mechanistically, ligation of Fc*γ*RIV promotes cellular activation (e.g., CD86 expression) and increases antigen presentation (28). We, and others, have shown that immune complexes consisting of antigen:IgG2a (or IgG2c) are preferentially taken up by splenic cDC subsets, which promotes increased T cell activation and proliferation (13, 21). Because blocking Fc*γ*RIV can ameliorate autoimmune and alloimmune IgG2a/c-mediated pathology, Fc*γ*RIV is an attractive therapeutic target.

Treatment with the anti-Fc*γ*RIV 9e9 antibody significantly reduced detectable Fc*γ*RIV on the surface of all splenic antigen presenting cell subsets that were analyzed. Unexpectedly, there was also reduced expression of Fc*γ*RIII and Fc*γ*RII (but not Fc*γ*RI) on different subsets. Thus, one must consider that some of the effects of 9e9 may be due to altering levels of Fc*γ*RIII and/or Fc*γ*RII. However, we show herein that Fc*γ*RIII^-/-^ mice have normal induction of alloimmunization by IgG2c. Also, we have shown previously that Fc*γ*RII is not sufficient for alloimmunization, as common *γ* chain knockout mice have not IgG2c-mediated enhancement of RBC alloimmunization (13). Thus, we reject the interpretation that decreased Fc*γ*RII and Fc*γ*RIII contributed to blockade of alloimmunization by 9e9.

Interestingly, passive immunization with IgG2c anti-HOD mAb induced higher levels of enhancement in Fc*γ*RIII^-/-^ than in FcγRIII^+/+^ mice. The reason for this difference is unclear, as Fc*γ*RIII^+/+^ is not typically considered inhibitory; however, it could be the result of decreased competition with Fc*γ*RIV for the common *γ* chain required for Fc*γ*R signaling or contributions of the background genetics (29), which may influence immune responses.

Blocking Fc*γ*RIV with 9e9 significantly reduced HOD RBC alloantibody levels following passive immunization with anti-HOD IgG2c mAb. However, throughout a 3-week time course, alloantibody levels did gradually increase. This may be attributed to the half-life of the 9e9 antibody, as only one infusion was given. Repeating these studies in Fc*γ*RI and Fc*γ*RIV knockout mice would address the limitations of using a blocking antibody; however, Fc*γ*R deficiency modulates expression of other Fc*γ*Rs (18, 30). Finally, it is worth noting that passive immunization with anti-HOD IgG2c mAb in 9e9 treated Fc*γ*RIII^-/-^ animals resulted in much lower levels of RBC alloantibodies, as compared to wild-type B6 recipients. Thus, although Fc*γ*RIII was not required for enhancing RBC alloantibody levels, it may still contribute to alloimmune responses; in particular, inhibiting both Fc*γ*RIII and Fc*γ*RIV simultaneously is required to prevent all such RBC alloantibody production (**Figure 3A**).

Polyclonal antibodies, such as human anti-D, contain a heterogenous mixture of IgG subclasses, consisting of multiple epitope specificities that may work in an additive, synergistic, or even an inhibitory, manner. Because the therapeutic anti-D used for immunoprophylaxis against Rh disease is derived by pooling material from multiple human volunteers, the efficacy of anti-D could depend on numerous variables, including donor-specific and antibody-specific characteristics. For example, a recent analysis of 23 such human monoclonal antibodies revealed that glycosylation profiles influenced biological activity (31), and IgG glycosylation can vary from human to human. Moreover, studies in mice highlight additional complexities that contribute to antibody-mediated immune responses, including affinity, the RBC antigen target and/or epitope, and RBC antigen copy number (13–15, 32–34). In addition to modulating immune enhancement or suppression, subclass can also dictate which effector pathways are employed (e.g., complement, FcRs, etc.) (35, 36). Thus, additional mechanistic investigations to elucidate how polyclonal anti-D prevents RhD alloimmunization will instruct the future development of human monoclonal anti-D antibodies, such that subclass, affinity, glycosylation, and epitope recognition can be fine-tuned to exert the desired function(s). Of note, human Fc*γ*RIIIA is the human ortholog to mouse Fc*γ*RIV (25, 37). Human IgG1 and IgG3, the main subclasses evaluated for monoclonal anti-D preparations, have high affinities for Fc*γ*RIIIA; however, this affinity is influenced by Fc*γ*RIIIA polymorphisms and the concentration of the antibody:antigen immune complexes (38). Thus, in addition to donor-specific and antibody-specific characteristics, recipient-specific characteristics will also need to be considered. In addition to the generalizable advantages of monoclonal antibody therapy (e.g., cost-effective, scalable, high specificity, lot-to-lot consistency), the substantial advances in antibody technology and increased understanding of immune function, now allow antibodies to be engineered to have increased affinity for the neonatal Fc receptor (FcRn), thereby facilitating an extended half-life to make passive immunization more durable. Moreover, antibodies can be engineered to inhibit antibody:FcRn interactions at the maternal-fetal interface, which is especially important for preventing hemolytic disease of the fetus and newborn mediated by alloantibodies to non-D RBC antigens (39, 40).

There are several limitations to the current studies. Although there is a clear reduction in alloantibody production upon blocking Fc*γ*RIV with 9e9, these studies would be strengthened by validating these findings in Fc*γ*RIV deficient animals or with another blocking antibody with a different binding site for Fc*γ*RIV. Additionally, passive immunization with anti-HOD IgG2c mAb followed by HOD transfusion leads to endogenous production of both IgM and IgG antibodies (13). Thus, while Fc-dependent Fc*γ*RIV ligation and signaling is blocked and signaling by IgG2c subclass antibodies is blocked, these antibodies may signal through other FcRs (e.g., FcmR) and modulate alloimmune responses (41). Finally, although the current analyses were limited to splenic antigen presenting cells, it is also likely that immune complex clearance also occurs in the liver.

The data presented herein demonstrate that Fc*γ*RIV, but neither Fc*γ*RIII nor Fc*γ*RI, is required for IgG2c-mediated enhanced alloimmunization to RBCs. These results, combined with our prior findings (13), support a model whereby passive immunization with an RBC-specific IgG2c antibody increases RBC alloantibody production to the corresponding antigen through Fc*γ*RIV ligation on splenic cDCs. To apply these mechanistic insights human anti-D, the future design of monoclonal antibodies should be tailored and optimized to leverage distinct subclass effector functions (12), Fc receptor expression patterns (41), and signaling (42).

## Data availability statement

The raw data supporting the conclusions of this article will be made available by the authors, without undue reservation.

## Ethics statement

The animal study was reviewed and approved by Columbia University Irving Medical Campus Institutional Animal Care and Use Committee (IACUC).

## Author contributions

AQ and KH designed the studies and experiments. AQ, AM, FD, MS, and MT set up experiments, collected and processed samples, and performed data analysis. All authors participated in data interpretation, revised the manuscript, and approved of the submitted version.

## Funding

These studies were supported in part by the National Institutes of Health R01HL135248 (KH) and P01HL132819 (JZ) by National Heart, Lung, and Blood Institute.

## Acknowledgments

The authors thank Drs. Steven L. Spitalnik and Eldad Hod for critical review of the manuscript.

## Conflict of interest

Although unrelated to the contents of this manuscript, KH has a sponsored research agreement with Alpine Immune Sciences. JZ is a consultant for Rubius Therapeutics and is the founder and CSO of Svalinn Therapeutics.

The remaining authors declare that the research was conducted in the absence of any commercial or financial relationships that could be construed as a potential conflict of interest.

## Publisher’s note

All claims expressed in this article are solely those of the authors and do not necessarily represent those of their affiliated organizations, or those of the publisher, the editors and the reviewers. Any product that may be evaluated in this article, or claim that may be made by its manufacturer, is not guaranteed or endorsed by the publisher.
