# Supplementary material for: FcγRIV is required for IgG2c mediated enhancement of RBC alloimmunization
Source: Front Immunol. 2022 Sep 13;13:972723. doi: 10.3389/fimmu.2022.972723 (PMC9519184; doi:10.3389/fimmu.2022.972723)
Supplement: Supplementary file 1 [file DataSheet_1.docx]

Fc$\gamma$RIV is required for enhancing RBC alloimmunization with IgG2c

**Supplemental Table**

**Supplemental Figures and Figure Legends**

**Supplemental Figure 1: Absolute numbers of each splenic antigen presenting cell subset.** B6 recipient mice received an infusion of 200ug of anti- Fc$\gamma$RIV clone 9e9, IgG isotype control, or PBS. Spleens were collected 30 minutes later and processed into single cell suspensions. Splenocytes were stained with antibodies to delineate cell subsets. The absolute number for each subset was calculated. Statistical analysis was performed with a one-way ANOVA with Tukey’s multiple comparisons test.

**Supplemental Figure 2: Pre-transfusion analysis of RBCs.** Whole blood was collected into 14% CPDA-1 from RBC donor animals, leukoreduced, labeled with CellTrace dye, and resuspended in PBS at 20% hematocrit. HOD-CellTrace-CFSE and B6-CellTrace Far Red were mixed at a 1:1 ratio. To determine the pre-transfusion ratio, an aliquot of the mixed RBCs were analyzed on a flow cytometer.

**Supplemental Figure 3: Inhibition of RBC Fc**γ**RIV prevents IgG2c-mediated production of IgM and IgG subclasses.** B6 recipient mice were passively immunized with 1ug of anti-HOD IgG2c followed by an RBC transfusion 2 hours later. Some animals received an infusion of 1ug of 9e9, an anti-FcγRIV blocking antibody, or IgG isotype control 30 minutes prior to passive immunization. Sera was collected weekly and analyzed for anti-HOD day 7 IgM, and day 14 IgG1, IgG2b, IgG2c, and IgG3 alloantibodies by flow crossmatch. Data are representative of 4 independent experiments with 4-5 mice per group. Statistical analysis was performed with a one-way ANOVA with Tukey’s multiple comparisons test, ****p<0.0001, ***p<0.001, **p<0.05, *p<0.05, ns = not significant.

**Supplemental Figure 4. HOD RBC clearance with IgG2c in FcγRIII^-/-^ and FcγRIII^+/+^ mice.** Recipient mice were passively immunized with 1ug of anti-HOD IgG2c followed by an RBC transfusion 2 hours later. Some animals received an infusion of 1ug of 9e9 or IgG isotype control 30 minutes prior to passive immunization. To assess RBC clearance, survival of HOD RBCs was determined in (A) FcγRIII^-/-^ and (B) FcγRIII^+/+^ recipient mice over 21 days.
